# Laparoscopic hepaticopancreaticoduodenectomy for synchronous intrahepatic cholangiocarcinoma and distal common bile duct tumors: a case report

**DOI:** 10.3389/fonc.2025.1665399

**Published:** 2025-11-10

**Authors:** Yuhui Fang, Dongdong Huang, Yuquan Chang, Qijie Luo, Ruiqin Huang, Kun He

**Affiliations:** 1 Guangdong Medical University, Zhanjiang, Guangdong, China; 2 Department of Hepatobiliary Surgery, Zhongshan City People’s Hospital, Zhongshan, Guangdong, China

**Keywords:** laparoscopic hepaticopancreaticoduodenectomy, surgery, case report, intrahepatic cholangiocarcinoma, bile duct tumor

## Abstract

**Background:**

Laparoscopic hepatopancreatoduodenectomy (LHPD) is a while very complex procedure for biliary malignancies combined with intrahepatic bile duct invasion, but there are few reports of related surgeries due to high postoperative complications and mortality. In this study, we report a case of tubular adenoma with high-grade intraepithelial neoplasia of the homologous left hepatic bile duct combined with the common bile duct.

**Case presentation:**

A 53-year-old female was admitted to the hospital with jaundice for one week. Imaging studies showed space-occupying lesions in both the left intrahepatic and common bile duct. We performed LHPD, and the patient was discharged on postoperative day 13 without bile and pancreatic fistula. Pathology confirmed tubular adenoma with high-grade intraepithelial neoplasia in both sites.

**Conclusion:**

LHPD can be an option for radical surgery in carefully screened patients with biliary malignancies with intrahepatic invasion.

## Background

Hepaticopancreaticoduodenectomy (HPD) is a high-risk surgical procedure performed to treat complex malignant tumors in the hepatobiliary and pancreatic regions. The extent of resection includes the liver, extrahepatic biliary system, and pancreaticoduodenum (1, 2). Although Takasaki et al. reported in 1980 that HPD could be a potential radical surgery for gallbladder cancer with invasion of the liver parenchyma or extrahepatic bile duct, research on its effectiveness for treating biliary tumors is still ongoing (2, 3). However, HPD is not widely accepted in Western countries because of its high postoperative morbidity and mortality rates (4). A North American cohort study revealed that the overall morbidity and mortality rates were significantly higher with HPD than with major hepatectomy (MH) and pancreaticoduodenectomy (PD) (87% vs. 51% vs. 52% for morbidity; 26% vs. 7.6% vs. 1.4% for mortality, respectively) (5). Owing to advancements in surgical procedures and accumulated experience, R0 resection after HPD for biliary tumors can significantly reduce mortality rates in patients with a sufficient residual liver volume and a sufficient functional liver remnant (FLR) greater than 40%. However, strict screening of suitable candidates is essential (5–7). Laparoscopic pancreaticoduodenectomy (LPHD) offers advantages over traditional approaches, including reduced trauma, less blood loss, a clearer surgical field, and fewer postoperative complications. It has been attempted in some larger surgical centers (8–11). A case in which LPHD was performed to treat synchronous tumors of the lower common bile duct and intrahepatic bile duct is reported here. Postoperative pathology revealed tubular adenoma with high-grade intraepithelial neoplasia.

## Case report

**Figure 1:**
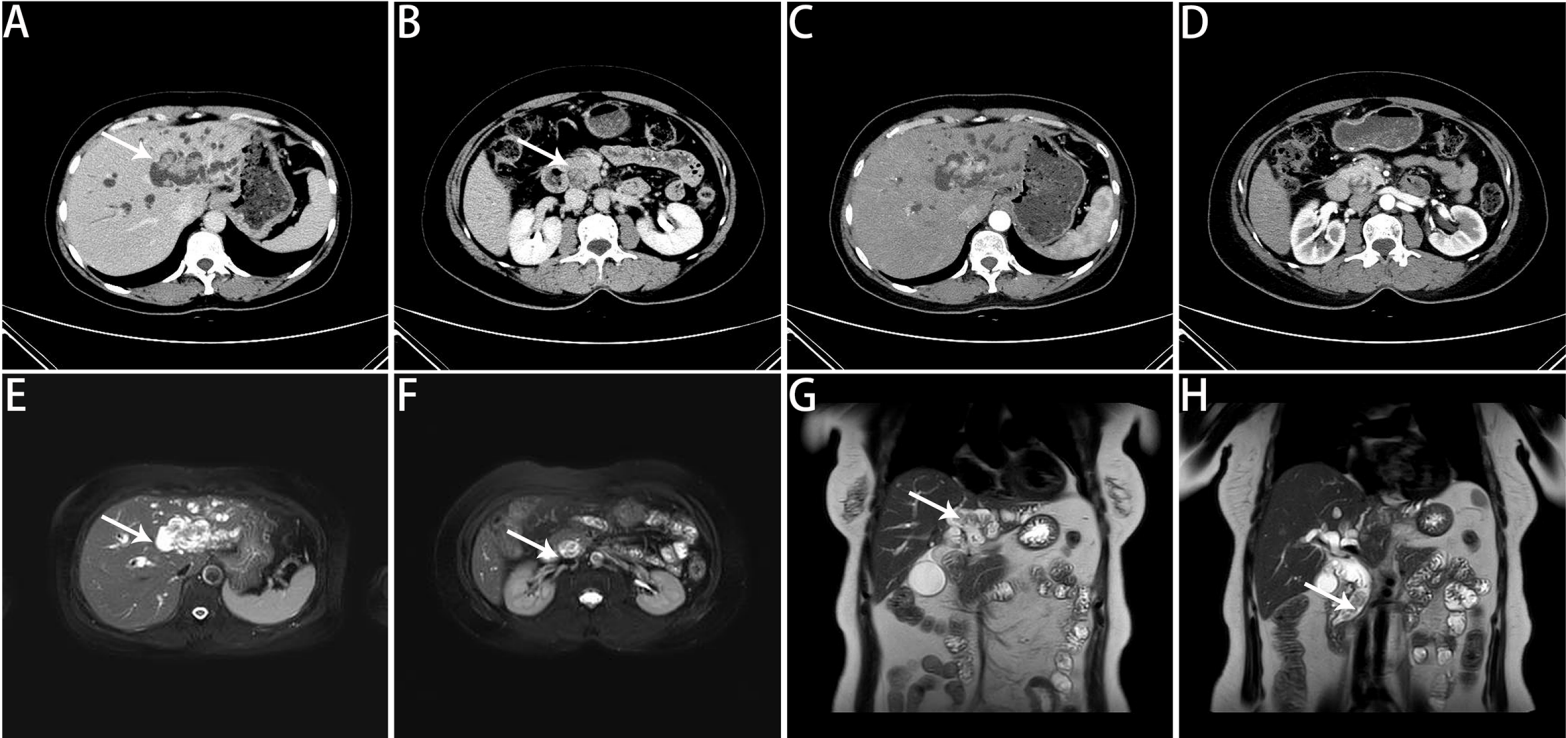
Preoperative imaging findings. **(A)** Noncontrast CT scan revealing multiple isodense nodules within the left intrahepatic bile ducts. **(B)** Noncontrast CT scan showing multiple isodense nodules within the distal common bile duct. **(C)** CE-CT in the arterial phase revealing space-occupying lesions with marked enhancement and irregular borders in the left intrahepatic bile ducts. **(D)** CE-CT in the arterial phase revealing markedly enhanced space-occupying lesions in the distal common bile duct. **(E)** MRCP image revealing multiple irregular space-occupying lesions in the left intrahepatic bile ducts. **(F)** MRCP image showing multiple irregular space-occupying lesions in the distal common bile duct. **(G)** MRCP coronal view showing multiple irregular space-occupying lesions in the left intrahepatic bile ducts. **(H)** MRCP coronal view showing multiple irregular space-occupying lesions in the distal common bile duct. The white arrows indicate lesions.

**Figure 2:**
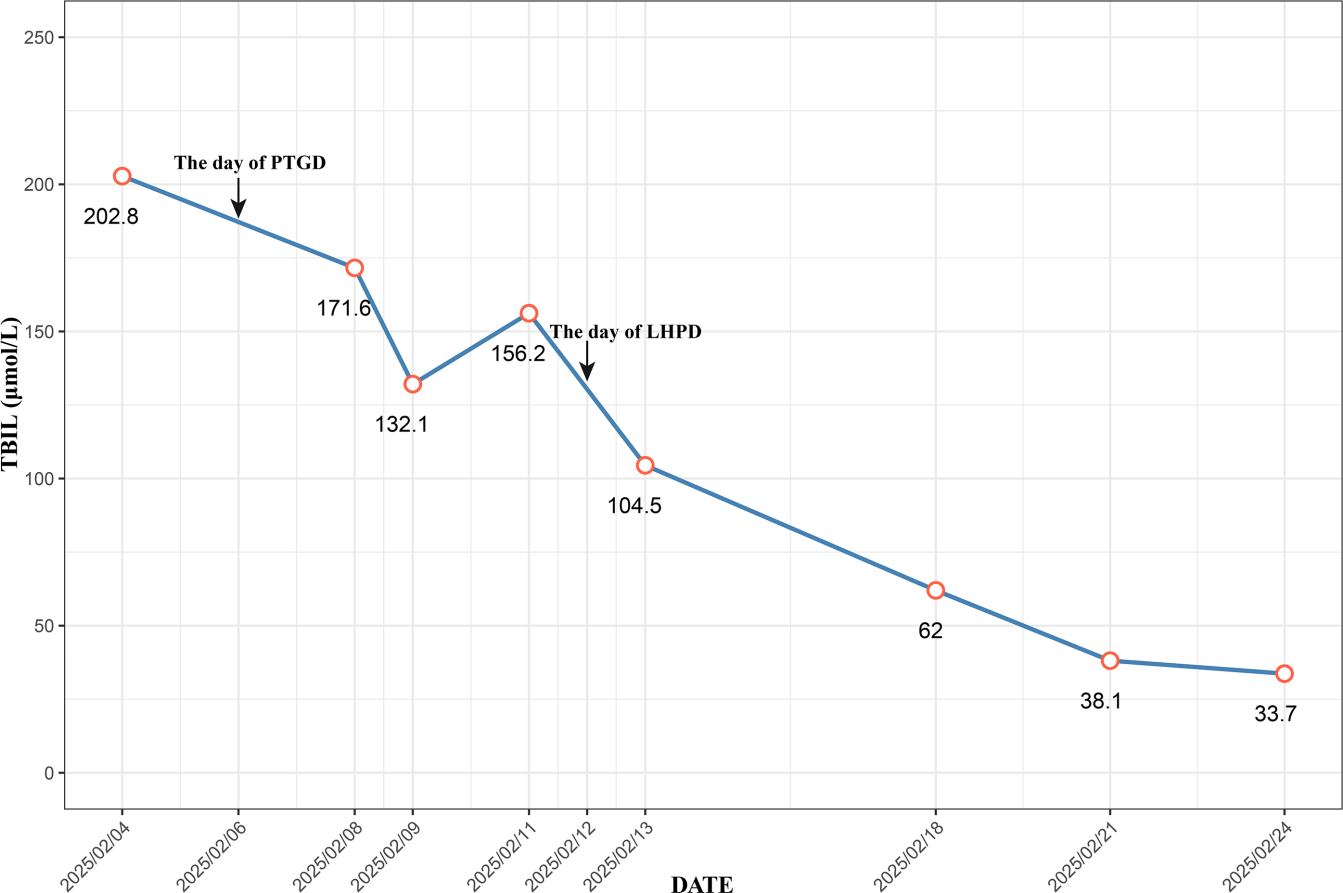
Trend of Bilirubin Levels During Hospitalization.

A 53-year-old female was admitted to Zhongshan People’s Hospital on February 3, 2025, due to jaundice that had persisted for more than one week. The patient had previously undergone laparoscopic total hysterectomy and adnexectomy. Physical examination revealed yellowing of the skin and sclera and tenderness in the right upper abdomen and subxiphoid region, with all other findings being negative. Preoperative laboratory tests revealed hepatitis B and significantly elevated levels of bilirubin, transaminases, and tumor markers (**Table 1**). Contrast-enhanced computed tomography (CE-CT) and magnetic resonance cholangiopancreatography (MRCP) revealed stenosis of the lower common bile duct lumen, local thickening of the bile duct wall and multiple enhancing space-occupying lesions, significant dilation of the left hepatic duct and intrahepatic bile duct lumen, and multiple irregular filling defects (**Figure 1**). On the basis of these findings, the preoperative diagnosis was intrahepatic cholangiocarcinoma combined with common bile duct carcinoma. Considering the patient’s significantly elevated total bilirubin, impaired preoperative liver function, and high risk of surgical bleeding, we performed ultrasound-guided percutaneous transhepatic gallbladder drainage (PTGD) to rapidly reduce biliary pressure and prevent the progression of acute liver failure. Postoperatively, the daily bile drainage volume was approximately 390 mL. After six days of drainage, the patient’s total bilirubin decreased to 156.2 μmol/L, representing a 77% reduction (**Figure 2**). On February 12, 2025, we performed LHPD.

**Table 1:** Preoperative blood laboratory parameters of patients.

Laboratory test	Result	Reference value
ALT(u/l)	141	7-40
AST(u/l)	194	13-35
TBIL(μmol/l)	202.8	2.0-20.4
ALB(g/l)	34.1	40-55
ALP(u/l)	631	50-135
CA19-9(u/ml)	98.2	0-34
CEA(ng/ml)	2.4	0-5

ALT, Alanine aminotransferase; AST, Aspartate aminotransferase; TBIL, Total bilirubin; ALB, albumin; ALP, Alkaline phosphatase; CA19-9:Carbohydrate Antigen19-9; CEA, Carcinoembryonic antigen.

**Figure 3:**
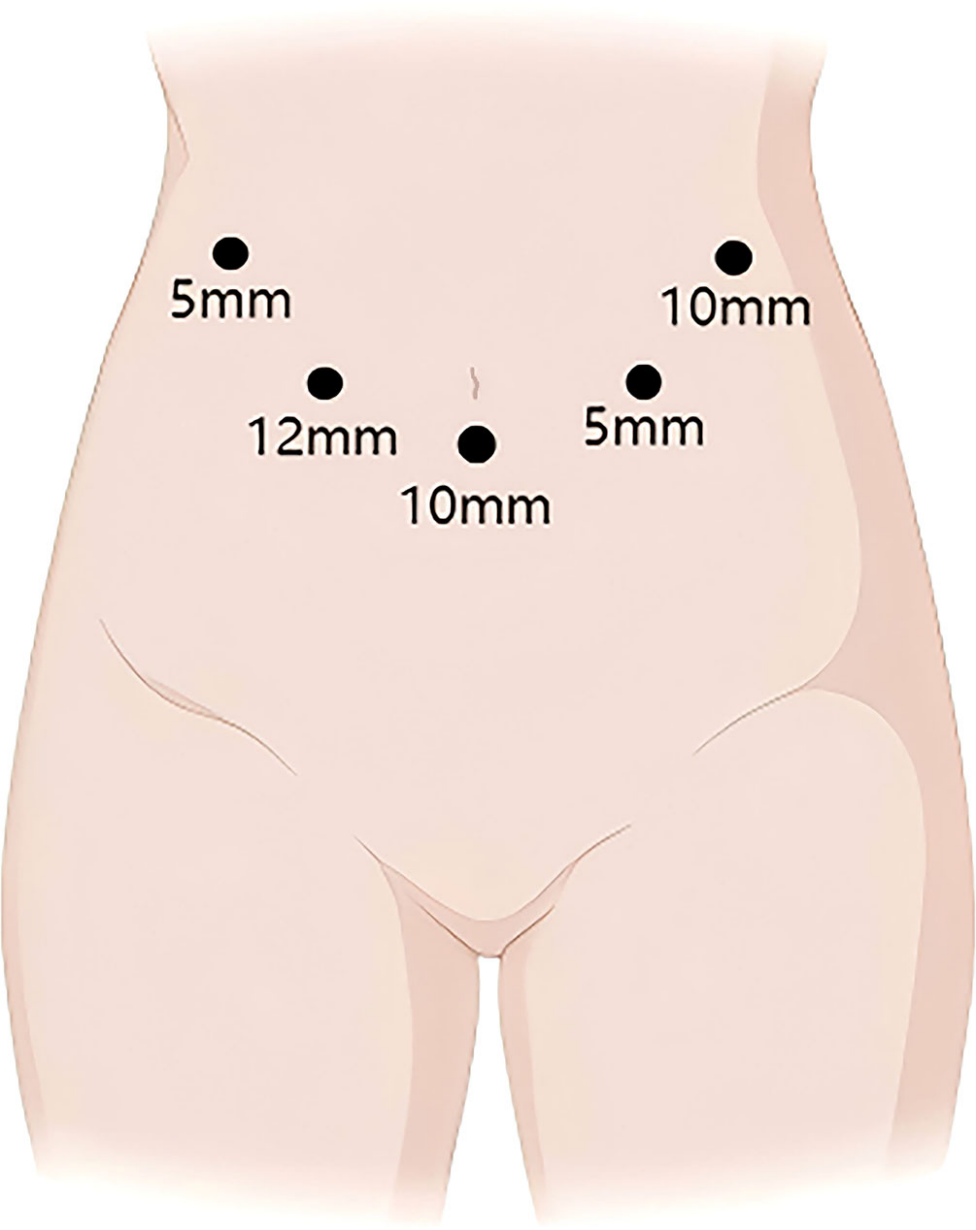
Distribution of laparoscopic trocar holes.

**Figure 4:**
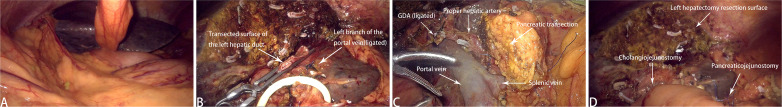
Intraoperative images. **(A)** Intraoperative laparoscopic overview. **(B)** Resected left hemihepatectomy sample. **(C)** Surgical field, including the Heidelberg triangle and the pancreatic transection surface. **(D)** Postoperative wound following LHPD.

The patient is placed in the supine split-leg position. The surgery was performed using the V-shaped 5-port technique (**Figure 3**). First, First, the gallbladder triangle was dissected and the gallbladder was removed. An ultrasonic scalpel was used to separate the falciform ligament, left coronary ligament, and hepatogastric ligament so that the left side of the liver could be elevated. The lymph nodes along the upper border of the pancreas and the hepatic hilar lymph nodes were dissected. The left hepatic artery, gastroduodenal artery, and left portal vein branch underwent ligation and transection. The ischemic segment of the left hemiliver was demarcated, and the liver parenchyma was incised, consequently exposing the segment VIb branch of the middle hepatic vein for subsequently dissection. The incision was continued cephalad to transect the hepatic parenchyma, thereby exposing the left hepatic duct. The origin of the left hepatic duct was clamped with double bulldog clamps and then transected, and no tumor was observed at the resection margin. The liver parenchyma was further divided up to the root of the left hepatic vein, and finally, the left hepatic vein was transected using an Endo GIA Stapler, indicating the completion of left hemiliver resection (**Figure 4B**). PD was performed using a venous approach. First, the gastrocolic ligament and adhesions of the posterior gastric wall were divided, the pancreas and duodenum were mobilized, and the abdominal aorta, inferior vena cava, left renal vein, and superior mesenteric artery root were surgically exposed. The right gastroepiploic artery and right gastric artery were divided, and then the gastric antrum and pancreatic neck were transected (**Figure 4C**). Finally, the jejunum was transected approximately 15 cm distal to the ligament of Treitz, the common hepatic duct was divided 1 cm below the confluence of the left and right hepatic ducts, and PD was completed.

The digestive tract was reconstructed using the Child’s method. The modified Blumgart technique was used for pancreaticojejunostomy—a stent was placed in the main pancreatic duct and the pancreatic duct was continuously sutured to the jejunum using 5–0 polydioxanone sutures. For choledochojejunostomy, an end-to-side anastomosis was created using 4–0 barbed sutures. The jejunum was anastomosed to the posterior gastric wall incision approximately 50 cm from the stoma using an Endo GIA Stapler, marking the completion of gastrojejunostomy (**Figure 4D**). The surgical field was inspected to ascertain the presence or absence of bleeding, and the specimen was removed for pathological analysis. The abdominal cavity was irrigated with warm distilled water, and an abdominal drainage tube was placed. The operation lasted 575 minutes, with an estimated blood loss of approximately 200 ml.

**Figure 5:**
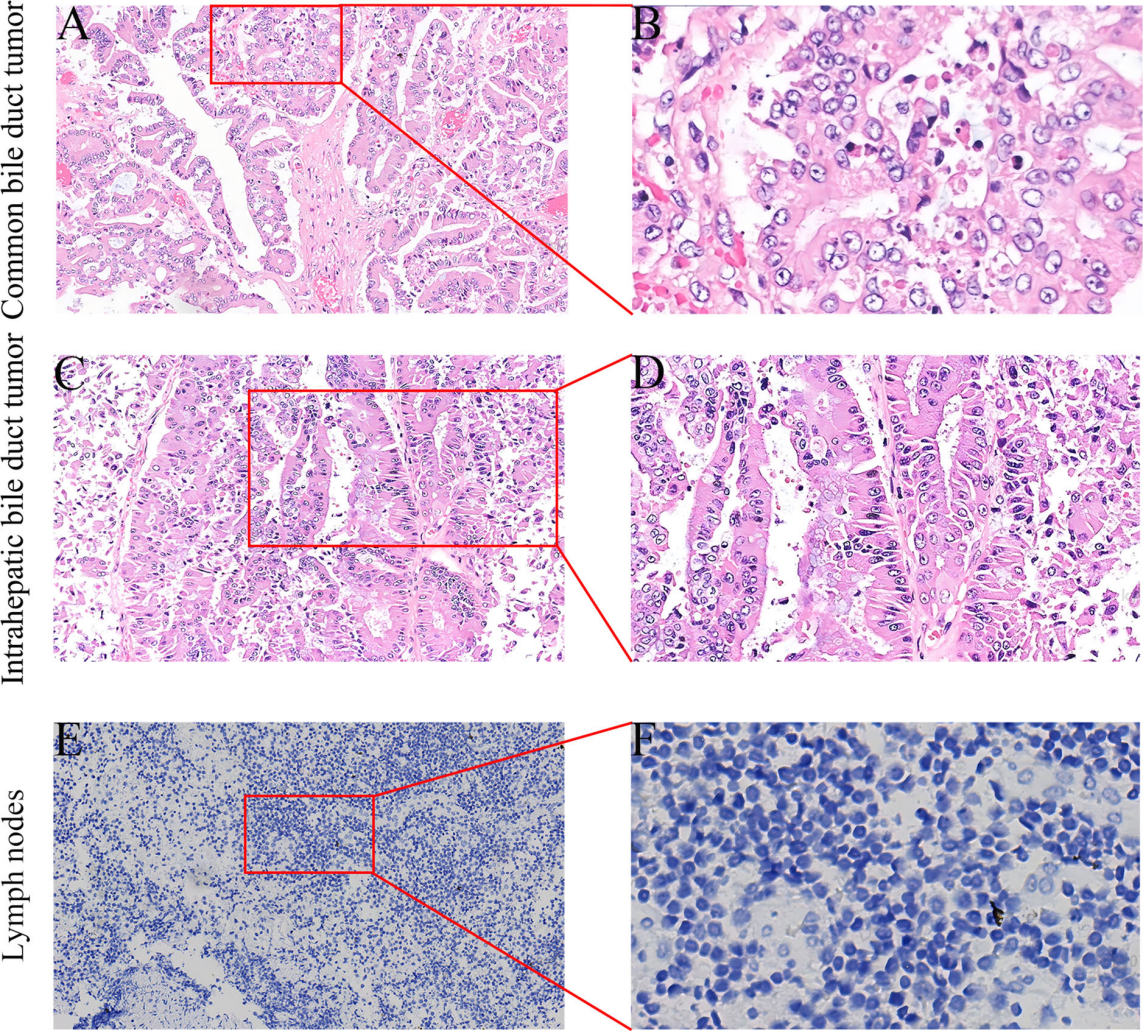
Postoperative specimen immunohistochemistry (IHC) results **(A-F)**. **(A)** HE staining of the common bile duct mass (×100). **(B)** Histologically, hyperplastic glands with a crowded arrangement and focal cribriform pattern are observed (×400). **(C)** HE staining of the left intrahepatic bile duct mass (×100). **(D)** Microscopically, the cells exhibit a columnar morphology with enlarged hyperchromatic nuclei, increased cytologic atypia, disorganized arrangement, and loss of polarity (×200). **(E)** Negative CK19 IHC staining (×100). **(F)** Microscopically, only blue-stained nuclei and clear cytoplasm are observed, with no high-grade intraepithelial neoplastic cells (×400).

After the operation, we conducted nursing monitoring for the patient, actively enhancing anti-infection treatment, nutritional support and correcting hypoproteinemia. We focused on monitoring changes in the volume and characteristics of the drainage fluid from the abdominal drainage tube and regularly repeated blood tests and abdominal CT scans to detect any fluid accumulation or infection in the surgical area. If fluid accumulation was detected, we promptly performed ultrasound-guided catheter drainage to prevent pancreatic fistula from eroding the blood vessels. The patient recovered smoothly postoperatively, with no signs of pancreatic leakage, biliary leakage, or liver failure. The patient was discharged on postoperative day 13. Immunohistochemistry revealed that the tubular adenoma with high-grade intraepithelial neoplasia in the lower common bile duct and left intrahepatic bile duct, CK19 (–). All the lymph nodes were not involved, and no tumor cells were found in the specimen resection margin (**Figure 5**).

## Discussion

HPD is a complex surgical intervention for malignant biliary tumors. Although its indications remain unclear, the most suitable cases are those involving advanced biliary malignancies with local hepatic invasion. The procedure is suitable for patients in whom R0 resection is feasible and who have no distant metastases (12, 13). However, owing to the high postoperative mortality rate, severe complications, and unclear survival benefits, many surgeons do not recommend this procedure (4, 5, 14). Although most literature reports a high postoperative mortality rate and poor survival prognosis, in experienced surgical centers, the postoperative mortality rate can be controlled below 10%. Moreover, the 5-year overall survival rate of patients with R0 resection is comparable to that of patients who have undergone extensive liver resection (5-year survival: 41% vs 40%, P = 0.328) (15). It is worth noting that all patients experienced varying degrees of postoperative complications. Because HPD involves the resection of the liver, gallbladder, pancreas, duodenum, and part of the stomach, it disrupts the continuity of the digestive tract. The reconstruction steps are intricate, increasing the risks of abdominal adhesions and ischemic anastomotic tissues. Most HPD studies have reported complications such as wound infection (4% - 41.7%), intra-abdominal infection (12% - 69.5%), bile leakage (5% - 50%), and pancreatic fistula (19% - 77%) (15–23). Liver failure caused by HPD is the most common cause of perioperative death. An American retrospective study of 480 HPD patients revealed that extended hepatic resection was significantly associated with an increased risk of postoperative mortality, with postoperative complication rates and in-hospital mortality rates as high as 87% and 18.2% (P < 0.001) (24). A study by Welch et al. also found that the incidence of liver failure after HPD surgery was as high as 56%, compared with only 14% after extensive hepatectomy. In this context, preoperative portal vein embolization (PVE) is recommended to induce compensatory hyperplasia of the non-resected liver lobe to increase the future liver remnant (FLR), improve surgical safety and feasibility, and reduce postoperative liver failure-related mortality. In addition, associating liver partition and portal vein ligation for staged hepatectomy (ALLPS) has been used to rapidly increase the volume of the remnant liver. However, its role in HPD remains unclear due to its high mortality and morbidity (5, 13).

Against this background, LHPD has gradually attracted people’s attention due to its minimally invasive characteristics and technical potential. Compared with open surgery, the application of minimally invasive surgery in major abdominal surgeries has significantly reduced the incidence of postoperative complications and mortality. Multiple studies revealed that compared with traditional open procedures, Laparoscopic pancreaticoduodenectomy(LPD) and laparoscopic liver resection (LLR) not only significantly reduce the average length of hospital stay and shortens the postoperative recovery time but also do not significantly affect long-term survival rates (3-year OS: LPD 59.1% vs. open pancreaticoduodenectomy [OPD] 54.3%, p = 0.33; 5-year OS: LLR 78.6% vs. open liver resection [OLR] 75.7%) (25–28). Existing LHPD literature reports indicate that surgical blood loss was controlled within 400–600 ml. No severe postoperative complications occurred. Only 2 cases of mild pancreatic fistula and 1 case of biliary fistula were reported after surgery, which were considered acceptable. Yao’s report emphasizes that the proficiency of surgeons in laparoscopic techniques is a key factor in reducing postoperative complications, particularly proficiency in LPD and LLR, and emphasizes the need for standardized surgical procedures (**Table 2**) (8).

**Table 2:** Comparison of complications between HPD and LHPD.

Author	Research type	Year	Country or Region	Number	HPD surgical approach	Intraoperative blood loss (ml)	Operative time (min)	Hospital stay (days)	Complication (Clavien-Dindo≥3 class) (%)	Wound infection	Intra-abdominal infection	PHLF	POPF (Grade B/C)(%)	Bile leakage	Postoperative mortality rate (%)	Survival prognosis
Aoki et al	retrospective study	2016	Japan	52	laparotomy	1360	832.5	N/A	36.5%	N/A	N/A	3.8%	77%	N/A	1.9%	5-year survival:44.5%
Dai et al	retrospective study	2017	Hong Kong, China	12	laparotomy	1800	850.5	22.5	N/A	41.7%	25%	16.7%	33.3%	8.3%	25%	5-year survival:27.8%
Welch et al	retrospective study	2019	America	23	laparotomy	N/A	521	18	87% (overall incidence)	4%	69.5%	56%	29%	50%	26%	N/A
D’Souza et al	retrospective study	2020	Europe	66	laparotomy	1000	520	23	50%	N/A	N/A	13.6%	19.7%	27.2%	15% (Death within 90 days)	3-year survival of CCA:65% 3-year survival of GBC:25%
Shimizu et al	retrospective study	2020	Japan	37	laparotomy	1000	866	52	51.4%	24%	49%	45.9%	30%	11%	5.4%	5-year survival:36.8%
Liu et al	retrospective study	2020	China	16	laparotomy	1215	515	18	62.5%	6.3%	12.5%	31.5%	43.8%	25%	12.5%	5-year survival:20%
Endo et al	retrospective study	2021	Japan	422	laparotomy	2015	438	60.3	40.5%	16.4%	12.1%	5.9%	27.3%	16.1%	5% (Death within 30 days)	N/A
Porcu et al	retrospective study	2023	Italy	6	laparotomy	N/A	412	23	66.7%	N/A	N/A	50%	33.3%	0	33.3% (Death within 30 days)	Median OS:21 months
Yoshimi et al	retrospective study	2023	Japan	54	laparotomy	1845	745	62	67%	N/A	N/A	37%	61%	22%	9.3%	5-year survival:31.1%
Atyah et al	retrospective study	2024	China	19	laparotomy	≥400 (>80% of patients)	N/A	N/A	36.8%	N/A	15.78%	N/A	36.85%	5.26%	5.26%	Median OS:17 months
Wu et al	retrospective study	2025	China	57	laparotomy	600 (77% of the patients)	350 (77% of the patients)	N/A	42.1%	8.7%	N/A	7%	26.3%	19.3%	1.7%	Median OS:13 months
Sugiura et al	retrospective study	2025	Japan	100	laparotomy	1842	668	35	82%	N/A	19%	11%	68%	17%	2%	5-year survival:40.7%
Kiritani et al	retrospective study	2025	Japan	45	laparotomy	1250	760	35	37.8%	N/A	N/A	15.6%	53.4%	13.3%	2.2% (Death within 90 days)	5-year survival:42%
Zhang et al	case report	2014	China	1	laparoscopic surgery	600	450	16	N/A	N/A	N/A	N/A	N/A	Yes	N/A	N/A
James et al	case report	2020	India	1	laparoscopic surgery	610	500	12	N/A	N/A	N/A	N/A	N/A	N/A	N/A	N/A
Lee et al	case report	2021	Korea	1	laparoscopic surgery	750	741	21	N/A	N/A	N/A	N/A	N/A	N/A	N/A	N/A
Yao	case report	2022	China	1	laparoscopic surgery	400	380	12	N/A	N/A	N/A	N/A	N/A	N/A	N/A	N/A
Wu et al	case report	2023	China	1	laparoscopic surgery	500	540	12	N/A	N/A	N/A	N/A	Yes (Grade A)	N/A	N/A	N/A
Wang et al	case report	2025	China	1	Robotic surgery	408	300	11	N/A	N/A	N/A	N/A	Yes (Grade A)	N/A	N/A	N/A

PHLF, Posthepatectomy Liver Failure; POPF, Postoperative Pancreatic Fistula.

Our successful implementation of HPD is based on three key factors. The first is patient selection criteria: patients with relatively localized left intrahepatic bile duct tumors, postoperative FLR > 40%, a low incidence of postoperative liver failure, and normal preoperative ICG and liver function tests, indicating the patient’s ability to tolerate extensive liver surgery. The second is surgical operation: Pancreatic fistula is the most frequent complication following HPD and primarily results from pancreaticojejunostomy during the PD procedure. The high incidence may be attributed to risk factors specific to biliary tract cancer patients, soft pancreatic texture, prolonged operative time, and postoperative hepatic dysfunction (29). In this case, the patient underwent pancreaticojejunostomy using the modified Blumgart technique. This method involves the U-shaped placement of sutures, which reduces the shear force of the suture while completely wrapping the jejunum around the pancreatic stump, preventing pancreatic fluid leakage and reducing the incidence of pancreatic fistula (30). To prevent anastomotic stenosis, we placed a stent in the pancreatic duct to drain pancreatic fluid and further reduce the risk of postoperative pancreatic fistula. Continuous sutures were used on the anterior and posterior walls of the bile duct-jejunal anastomosis to reduce the risk of postoperative bile fistula. However, the mucosa of the left hepatic duct and common hepatic duct resection margins were normal. To reduce the risk of postoperative biliary stenosis, we retained part of the common hepatic duct and did not choose the right hepatic duct for anastomosis. In addition, we carefully ligated the blood vessels that are prone to bleeding due to pancreatic fistula (such as the gastrocolic trunk, dorsal pancreatic artery, gastroduodenal artery, and inferior pancreaticoduodenal artery) to prevent bleeding caused by the erosion of blood vessels by pancreatic fistula.

The limitation of this case lies in the fact that intraoperative frozen pathological examination was not performed on the resection margins of the left hepatic duct and common hepatic duct. Instead, based on the intraoperative observation that the mucosal morphology of the resection margins of the left hepatic duct and common hepatic duct was normal, and combined with preoperative imaging findings, it was judged that the left intrahepatic bile duct tumor and the lower segment tumor of the common bile duct did not invade the bile duct resection margins. This empirical speculation carried a certain degree of risk. However, ultimately, the patient achieved an R0 resection. Postoperatively, the patient did not develop serious complications and recovered well.

Overall, integrating the literature and the experience of this case, the following key aspects should be emphasized when performing LHPD: Firstly, strict patient selection criteria are required. This includes evaluating the FLR, conducting ICG tests, assessing the preoperative liver function, and determining whether R0 resection can be achieved, etc., in order to reduce the surgical risk (31). For patients with severe preoperative jaundice, percutaneous biliary drainage can be used to reduce bilirubin. For patients with insufficient FLR, PVE can be considered. Secondly, due to the complexity of the surgery, the operating surgeon should have extensive experience in LPD and LLR. Precise handling of areas such as choledochojejunostomy or pancreaticojejunostomy is necessary to reduce the risk of complications such as pancreatic fistula and ensure the safety of the surgery. In conclusion, LHPD is feasible and safe in experienced surgical centers and among strictly selected patients.

## Conclusion

For carefully selected patients with biliary tract malignancies and intrahepatic invasion, experienced surgeons can perform LHPD as a radical surgical treatment for such patients.

## Data Availability

The original contributions presented in the study are included in the article/supplementary material. Further inquiries can be directed to the corresponding author.
